# Mutation of *SlSBPASE* Aggravates Chilling-Induced Oxidative Stress by Impairing Glutathione Biosynthesis and Suppressing Ascorbate-Glutathione Recycling in Tomato Plants

**DOI:** 10.3389/fpls.2020.565701

**Published:** 2020-12-22

**Authors:** Meiling Wang, Fei Ding, Shuoxin Zhang

**Affiliations:** ^1^ School of Life Sciences, Liaocheng University, Liaocheng, China; ^2^ College of Forestry, Northwest A&F University, Yangling, China

**Keywords:** ascorbate, glutathione, chilling stress, oxidative stress, reactive oxygen species, SBPase, tomato

## Abstract

Sedoheptulose-1,7-bisphosphatase (SBPase) is a crucial enzyme for photosynthetic carbon assimilation in the Calvin-Benson cycle. Previous studies have shown that overexpression of SBPase is advantageous to chilling tolerance in plants; however, the mechanisms of SBPase acting in the improvement of chilling tolerance remain largely unknown. In the present study, we aimed to uncover the essential role of SBPase in the response of tomato plants to oxidative stress induced by low temperature. To fulfill that, we performed an array of comparative studies between *slsbpase* mutant plants that we previously generated using CRISPR/Cas9 genome editing system and their wild-type counterparts under chilling stress. It was observed that following a 24 h chilling treatment, *slsbpase* mutant plants accumulated higher levels of reactive oxygen species (ROS) than wild-type plants and consequently, more severe lipid peroxidation occurred in *slsbpase* plants. Activity assay of antioxidant enzymes showed that mutation in *SlSBPASE* significantly decreased activities of peroxidase (POD) and ascorbate peroxidase (APX), but surprisingly did not significantly alter activities of superoxide dismutase (SOD) and catalase (CAT) under the chilling condition. Notably, mutation in *SlSBPASE* reduced the contents of total ascorbate (AsA) and total glutathione (GSH) and suppressed the recycling of AsA and GSH in chilling-stressed tomato plants. In addition, activities of two GSH biosynthetic enzymes (gamma-glutamylcysteine synthetase and glutathione synthetase) and transcript abundance of their coding genes (*GSH1* and *GSH2*) were markedly reduced in *slsbpase* mutant plants in comparison with those in wild-type plants under chilling stress. Furthermore, exogenous GSH remarkably mitigated chilling damage in *slsbpase* plants. Collectively, these results support that mutation in *SlSBPASE* aggravates chilling-induced oxidative stress by suppressing GSH biosynthesis and AsA-GSH recycling and suggest that SBPase is required for optimal response to chilling stress in tomato plants. The findings also shed light on the idea to mitigate chilling-induced damages by genetically manipulating a photosynthetic enzyme in plants.

## Introduction

Cold stress impairs plant growth and development and severely reduces crop productivity (Hussain et al., 2018). Effects of cold stress on plants are multifaceted, but the most pronounced one is the intensive generation of reactive oxygen species (ROS; Foyer and Shigeoka, 2011; Wang et al., 2020). The excessive ROS inevitably results in oxidative damages to a diversity of macromolecules, including proteins, nucleic acids, carbohydrates, and lipids in plants (Apel and Hirt, 2004; Gill and Tuteja, 2010). Thus, the resistance to cold stress is, to a large extent, dependent on plants’ capability of detoxifying ROS. During evolution, plants have developed several mechanisms to minimize the adverse effects of ROS. Plants mainly deploy a concerted antioxidant network in response to elevated ROS levels. Multiple antioxidant enzymes and non-enzymatic compounds are engaged in this network. The common antioxidant enzymes consist of peroxidases (POD), ascorbate peroxidases (APX), superoxide dismutases (SOD), catalases (CAT), glutathione reductases (GR), and dehydroascorbate reductases (DHAR). The non-enzymatic antioxidant compounds mainly include low-molecular-weight and water-soluble compounds, such as ascorbate (AsA), glutathione (GSH), polyamines, among others (Yarmolinsky et al., 2014).

Plants also rely on the ascorbate-glutathione (AsA-GSH) cycle, which has been recognized as one effective antioxidant mechanism, to eliminate excessive ROS (Foyer and Halliwell, 1976). The AsA-GSH cycle operates in different subcellular locations, such as cytosol, chloroplast, mitochondria, and peroxisome. The cycle involves several components, including four antioxidant enzymes, APX, monodehydroascorbate reductase (MDHAR), DHAR, and GR, and two antioxidant compounds, AsA and GSH. Both AsA and GSH are potent antioxidative compounds, and their redox state is critical for the detoxification of ROS and chilling tolerance (Szarka et al., 2012). The recycling of AsA and GSH is mainly accomplished through the concerted action of the four antioxidant enzymes in the AsA-GSH cycle. These enzymes are thus important in maintaining the redox state of AsA and GSG (Hasanuzzaman et al., 2019).

Chloroplasts harbor light-dependent reactions and carbon-fixing reactions of photosynthesis are considered as one of the major sources of ROS production under adverse conditions. Under low temperature stress, the electron transport chain is often over-reduced and electrons are passed on to molecular oxygen, which gives rise to the formation of ROS (Mittler, 2002; Asada, 2006). The Calvin-Benson cycle is a well-established carbon-fixation pathway, in which a series of sequential steps are catalyzed by multiple enzymes, including sedoheptulose-1,7-bisphosphatase (SBPase). Prior studies on SBPase support that this enzyme is sensitive to chilling temperatures (Hutchison et al., 2000; Ding et al., 2017c). The perturbation of SBPase by chilling stress inhibits carbon fixation and may reduce the utilization of electrons produced by the light reactions, aggravating the generation of ROS. In addition to its well documented functions in photosynthesis and growth (Raines et al., 2000; Olçer et al., 2001; Driever et al., 2017), SBPase has been implicated in the protection of plants against several environmental stress factors, including salinity and high temperature (Feng et al., 2007a,b; Ding et al., 2016, 2017c); however, it is not clear how SBPase acts in the alleviation of chilling stress in plants.

Tomato is an important horticultural crop that is widely cultivated as sources of vitamins and other nutrients. Low temperature is considered as a major environmental stress threatening tomato productivity (Zushi et al., 2012; Ding et al., 2017b). In a previous study, we showed that the expression of the tomato gene *SlSBPASE* was induced by low temperature and its overexpression enhanced tomato growth and chilling tolerance (Ding et al., 2016). However, the mechanisms of SBPase mitigating chilling stress in plants remain largely unexplored. In this study, we made an array of comparisons of ROS accumulation, oxidative stress, and antioxidant capacity between *slsbpase* mutant plants generated previously *via* CRISPR/Cas9 gene editing system and their wild-type counterparts under the chilling condition. We have particularly investigated the potential impact of SBPase on GSH biosynthesis and AsA-GSH recycling in tomato plants under low temperature stress. The principal goal of this study was to provide specific evidence for SBPase, as a key photosynthetic enzyme, functioning to reduce chilling-triggered oxidative stress in tomato plants.

## Materials and Methods

### Plant Materials and Treatment

SBPase knockout mutant plants (*slsbpase*) that we previously generated (Ding et al., 2018a) and wild-type tomato plants (*Solanum lycopersicum* L. “Micro-Tom”) were used as plant materials in this study. In our previous study, we screened one homozygous mutant line with 1-bp deletion in the second exon of *SlSBPASE* and the transgenic line showed just residual activity of SBPase (Ding et al., 2018a). Seeds harvested from T1 transgenic plants were used in the present study. Seeds of T1 mutant plants and wild-type plants were germinated and grown in plastic pots containing peat and vermiculite at a volume ratio of 3/1. All plants were cultivated in a controlled growth chamber with CO_2_ level 400 μmol mol^−1^, photon flux density 400 μmol m^−2^ s^−1^, day/night temperature 25/20°C, relative humidity 65%, and light/dark cycle 14/10 h. After the fourth leaf emerged, tomato seedlings were subjected to chilling treatment at 4°C during 24 h. For GSH feeding experiment, tomato seedling leaves were sprayed either with H_2_O or 5 mM GSH for 3 consecutive days prior to chilling treatment. Following treatment, leaves were collected and immediately frozen in liquid nitrogen for further analysis. Chemicals used in this study were purchased from Sigma-Aldrich unless otherwise stated.

### Measurement of Photosynthesis

Photosynthesis measurements were performed on the third leaves of tomato plants using a LI-6400 photosynthesis system (LI-COR Biosciences, United States). The measurements were conducted under the controlled condition, with light density being 600 μmol m^−2^ s^−1^ and CO_2_ being 400 μmol mol^−1^.

### Determination of Sucrose Content

The third leaves were detached from different groups of tomato plants at the end of day for extraction and measurement of sucrose. The measurements were conducted as described previously (Stitt et al., 1989).

### Quantification of H_2_O_2_ and O_2_·^−^

Following a 24 h chilling treatment, tomato leaves were collected for the investigation of H_2_O_2_ accumulation and O_2_·^−^ production. Quantification of H_2_O_2_ was conducted by recording the absorbance of Ti-H_2_O_2_ complex at 410 nm as described previously (Patterson et al., 1984). The production of O_2_·^−^ was determined by following a previous protocol (Jabs et al., 1996).

### Determination of Malonaldehyde Content

Following a 24 h chilling treatment, tomato leaves were collected for the determination of malondialdehyde (MDA) level. Determination of MDA content was done as in a previous work (Ding et al., 2017a). Briefly, MDA was extracted with trichloroacetic acid and the absorbance measured at 532 and 600 nm was used to calculate MDA content.

### Measurement of Electrolyte Leakage

Following a 24 h chilling treatment, leaves of wild-type plants and mutant plants were detached for measurement of electrolyte leakage as previously described (Ding et al., 2018b).

### Enzyme Activity Assay

Leaf samples were collected after a 24 h chilling treatment and the enzymes were extracted with potassium phosphate buffer (50 mM, pH 7.5). After centrifugation, the supernatant was used to determine the antioxidant enzyme activities (Shi et al., 2007; Wang et al., 2019). To determine SOD activity, the photochemical reduction of nitro blue tetrazolium was used by measuring the absorbance at 560 nm (Beauchamp and Fridovich, 1971). For CAT activity determination, the reduction of absorbance at 240 nm was used (Cakmak and Marschner, 1992). To determine POD activity, the increase in absorbance at 470 nm was used (Nickel and Cunningham, 1969; Ding et al., 2017c). For APX activity determination, the change in absorbance at 290 nm was used (Nakano and Asada, 1981). To determine DHAR and MDHAR activities, the increase in absorbance at 265 and 340 nm was used, respectively (Nakano and Asada, 1981). To determine GR activity, the rate of decrease in the absorbance of NADPH at 340 nm was used (Foyer and Halliwell, 1976). To determine the activities of the enzymes gamma-glutamylcysteine synthetase (γ-ECS) and glutathione synthetase (GS), leaf samples from different treatments were collected and the enzymes were extracted with potassium phosphate buffer (50 mM, pH 7.0). γ-ECS activity was determined as described previously (Rüegsegger and Brunold, 1992). GS activity was determined according to a published method (Cobbett et al., 1998).

### Determination of Transcript Abundance by Quantitative Real-Time PCR

*GSH1* and *GSH2* gene transcript abundance was investigated by quantitative real-time PCR. Total RNA was extracted from different leaf samples and was used for cDNA synthesis. The determination was performed using a Premix Ex Taq kit (TaKaRa, China) according to the manufacturer’s instructions. Each real-time PCR reaction was performed on iQ5 Multicolor Real-Time PCR Detection System (BIO-RAD, United States). The primers used for *GSH1* were 5'-CCTCAGCACACCAAAATCCT-3' (forward) and 5'-GCTTTGTGCCTTGGCTCTAGT-3' (reverse). The primers used for *GSH2* were 5-AGTGGAAAGCTAGGCTGCTG-3' (forward) and 5'-TCATCCAAGCTCCACAACCC-3' (reverse).

### Determination of AsA and GSH Contents

Ascorbate content was measured according to a previous protocol (Sarker and Oba, 2018). About 0.1-mg leaf samples were extracted with 6% (v/v) HClO_4_. After centrifugation, the absorbance of the supernatant was measured at 265 nm in sodium acetate buffer and the absorbance of that solution following incubation with AsA oxidase (0.5 units) was also recorded at 265 nm. The difference in the absorbance was used to calculate AsA content. Measurement of GSH and GSSG contents was done as previously described (Griffith, 1980). The decomposition of 5,5′-dithiobis-(2-nitrobenzoic acid; DTNB) monitored at 412 nm was recorded to calculate total GSH. For the measurement of GSSG, 2-vinylpyridine was used to mask GSH.

### Statistical Analysis

Three biological repetitions were used for each experiment in this study, and the mean values were presented. The difference among treatments was compared by running Duncan’s multiple range test and value of *p* < 0.05 represents significant difference.

## Results

### Mutation in *SlSBPASE* Suppresses Photosynthetic Carbon Assimilation

**Figure 1:**
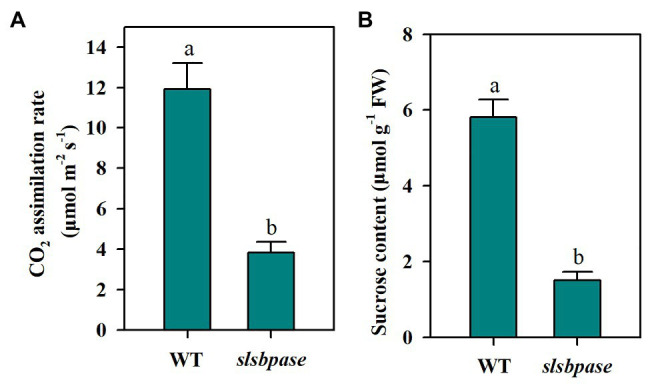
Decreased CO_2_ assimilation rate **(A)** and sucrose accumulation **(B)** as a consequence of *SlSBPASE* mutation in tomato T2 plants. The results are presented as mean values ± SDs from three independent experiments, and different letters on top of each column represent significantly different values at *p* < 0.05.

As a Calvin-Benson cycle enzyme, SBPase plays an important role in carbon assimilation and plant growth. In a previous study, we introduced a mutation in the *SlSBPASE* gene using a CRISPR/Cas9 genome editing system (Ding et al., 2018a). In the present study, we first tested whether *slsbpase* T2 plants showed similar changes in photosynthesis and sucrose accumulation as *slsbpase* T1 plants. We observed that as *slsbpase* T1 plants did (Ding et al., 2018a), *slsbpase* T2 plants also exhibited a remarkable decrease in carbon assimilation rate, in comparison with the wild-type plants (Figure 1A). Consistent with reduced carbon assimilation, *slsbpase* T2 plants showed a dramatic inhibition of sucrose accumulation (Figure 1B). These results confirm that *SlSBPASE* knockout mutation severely impairs photosynthetic carbon assimilation.

### Mutation in *SlSBPASE* Increases ROS Accumulation and Aggravates Lipid Peroxidation

**Figure 2:**
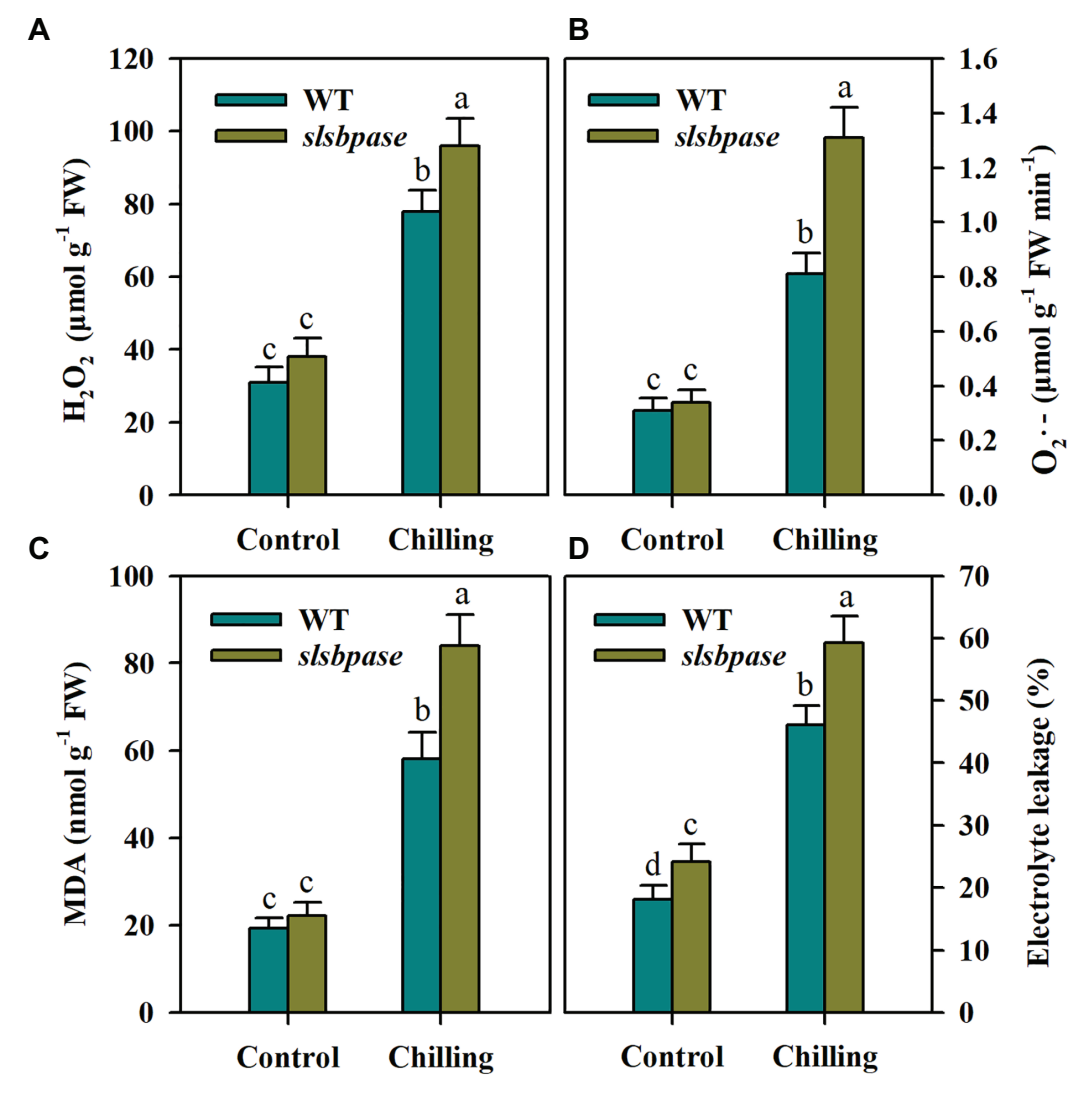
Reactive oxygen species (ROS) accumulation and lipid peroxidation as affected by *SlSBPASE* mutation in chilling-stressed tomato plants. Lipid peroxidation was assessed by measuring malondialdehyde (MDA) content and electrolyte leakage. **(A)** H_2_O_2_; **(B)** O_2_·^−^; **(C)** MDA content; and **(D)** electrolyte leakage. At the fourth-leaf stage, *slsbpase* mutants and wild-type plants were subjected to chilling stress for a period of 24 h. Following that, leaves from different groups were sampled for the quantification of ROS and measurement of lipid peroxidation. The results are presented as mean values ± SDs from three independent experiments, and different letters on top of each column represent significantly different values at *p* < 0.05 among treatments.

To investigate the impact of *SlSBPASE* mutation on oxidative stress triggered by chilling stress in tomato plants, we first measured the production of H_2_O_2_ and O_2_·^−^ in chilling-stressed tomato leaves. Under control conditions, *slsbpase* mutant plants accumulated slightly more H_2_O_2_ and O_2_·^−^ than wild-type plants. Following 24 h chilling stress, accumulations of H_2_O_2_ and O_2_·^−^ were sharply increased in all examined plants; however, the levels of H_2_O_2_ and O_2_·^−^ were significantly different between plants with impaired SBPase and wild-type plants, with more ROS being produced in loss-of-function SBPase plants (Figures 2A,B).

Lipid peroxidation of cell membranes is a common consequence of low temperature stress and is often used to assess cold damage to plants. We thus measured MDA content and electrolyte leakage to examine the membrane integrity in chilling-stressed *slsbpase* mutants and wild-type plants. It was observed that electrolyte leakage and MDA level were both dramatically enhanced by chilling stress in tomato plants. Nonetheless, *slsbpase* mutant plants accumulated more MDA and showed higher electrolyte leakage than wild-type plants under low temperature conditions (Figures 2C,D). Therefore, these results imply that SBPase may contribute to the mitigation of chilling-triggered lipid peroxidation.

### Mutation in *SlSBPASE* Does Not Significantly Alter Activities of SOD and CAT Under Chilling Stress

**Figure 3:**
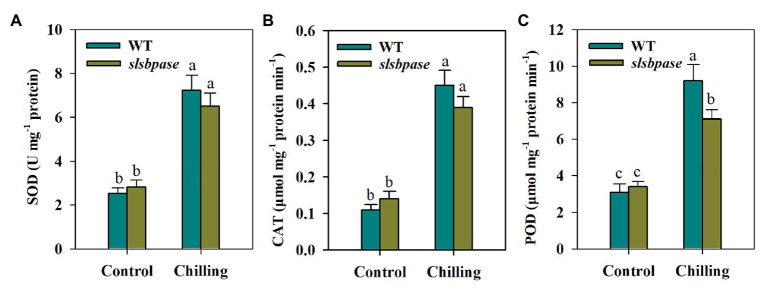
Activities of antioxidant enzymes were differentially affected by *SlSBPASE* mutation in chilling-stressed tomato plants. **(A)** Superoxide dismutase (SOD); **(B)** catalase (CAT); and **(C)** peroxidase (POD). At the fourth-leaf stage, *slsbpase* mutants and wild-type plants were subjected to a 24 h chilling stress. Following that, leaves from different groups were sampled for the determination of enzyme activities. The results are mean values ± SDs from three independent experiments, and different letters on top of each column represent significantly different values at *p* < 0.05 among treatments.

Antioxidant enzymes are key to redox homeostasis in plants, so we next evaluated the impact of *SlSBPASE* mutation on antioxidant enzymes in tomato plants by determining their activities. The results showed that chilling stress dramatically boosted activities of antioxidant enzymes in both *slsbpase* mutants and wild-type plants; however, it was found that SOD and CAT activities displayed no significant differences between wild-type plants and *slsbpase* mutants under the chilling condition. Different from CAT and SOD, POD activity was significantly decreased by mutagenesis of *SlSBPASE* (Figure 3).

### Mutation in *SlSBPASE* Negatively Impacts the AsA-GSH Cycle

**Figure 4:**
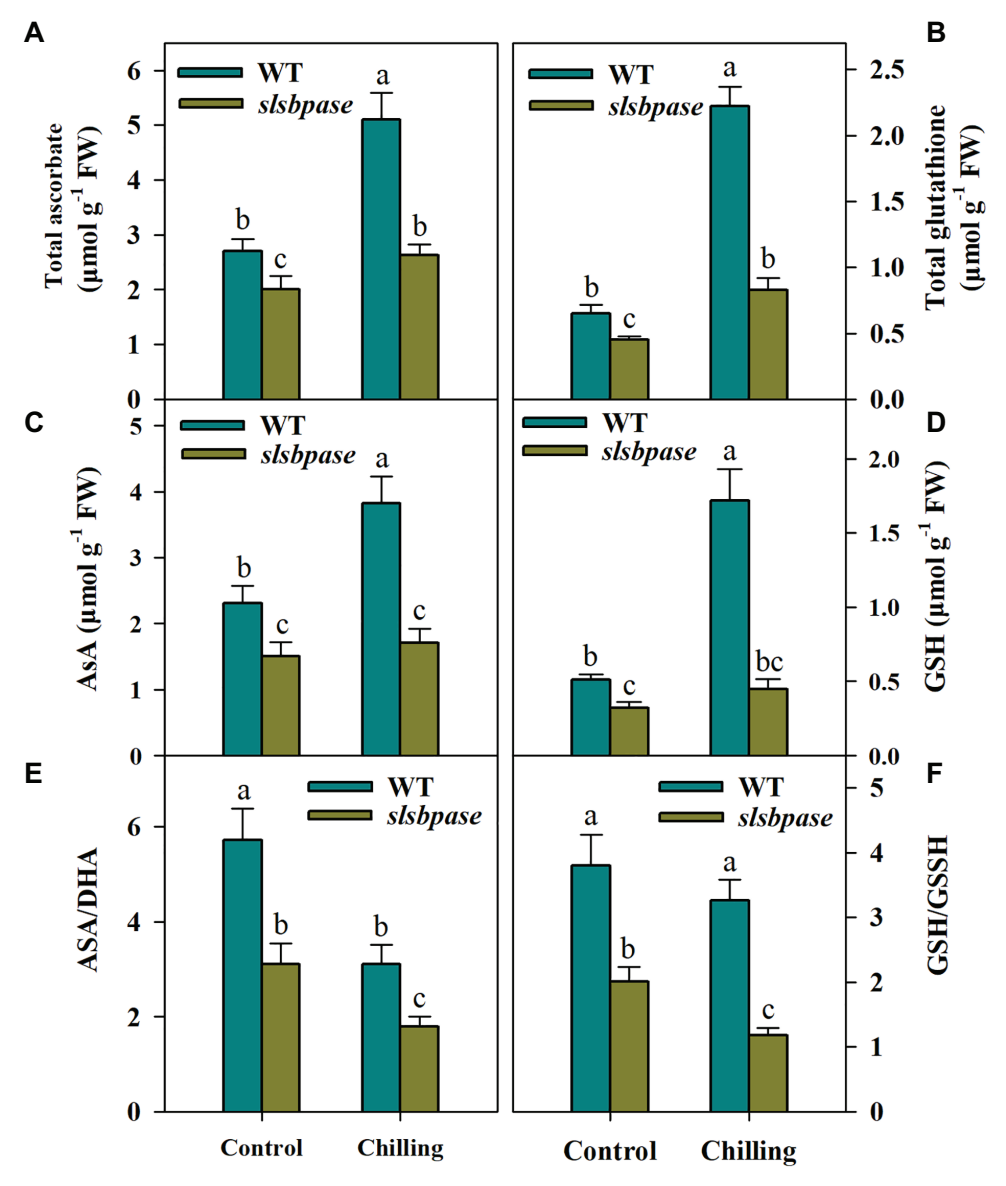
Levels of ascorbate (AsA) and glutathione (GSH) in the AsA-GSH pathway as affected by *SlSBPASE* mutation in chilling-stressed tomato plants. **(A)** Total AsA; **(B)** total GSH; **(C)** AsA; **(D)** GSH; **(E)** AsA/DHA ratio; and **(F)** GSH/GSSH. At the fourth-leaf stage, *slsbpase* mutants and wild-type plants were subjected to a 24 h chilling stress. Following that, leaves from different groups were sampled for the determination of AsA and GSH contents. The results are mean values ± SDs from three independent experiments, and different letters on top of each column represent significantly different values at *p* < 0.05 among treatments.

The Ascorbate-glutathione cycle plays a central role in the regulation of redox balance in plants under unfavorable environmental conditions; we thus investigated the potential impact of *SlSBPASE* mutation on the AsA-GSH cycle. Both AsA and GSH are well known as antioxidants and are key components in the AsA-GSH cycle. To find out whether *SlSBPASE* mutation affected the accumulation of AsA and GSH under chilling stress, we measured the levels of total AsA and total GSH as well as their respective reduced form. After chilling stress was imposed, total AsA and GSH contents in both mutant and wild-type plants increased markedly. However, *slsbpase* mutants produced considerably less AsA and GSH than their wild-type counterparts under chilling stress (Figures 4A–D). Also, under control conditions, contents of AsA and GSH were also significantly lower in *slsbpase* mutant plants that those in wild-type plants, suggesting that loss of SBPase affects metabolism of these two compounds.

AsA/DHA and GSH/GSSH ratios are critical for redox status in plants under various stresses. We then assessed the changes of these ratios in wild-type and *slsbpase* plants. It was observed that AsA/DHA and GSH/GSSH ratios in wild-type plants were higher than those in *slsbpase* mutants under both temperature conditions (Figures 4E,F), suggesting that SBPase may engaged in the redox regulation in chilling-stressed tomato plants.

### Mutation in *SlSBPASE* Negatively Affects Activities of Key Enzymes in the AsA-GSH Cycle

**Figure 5:**
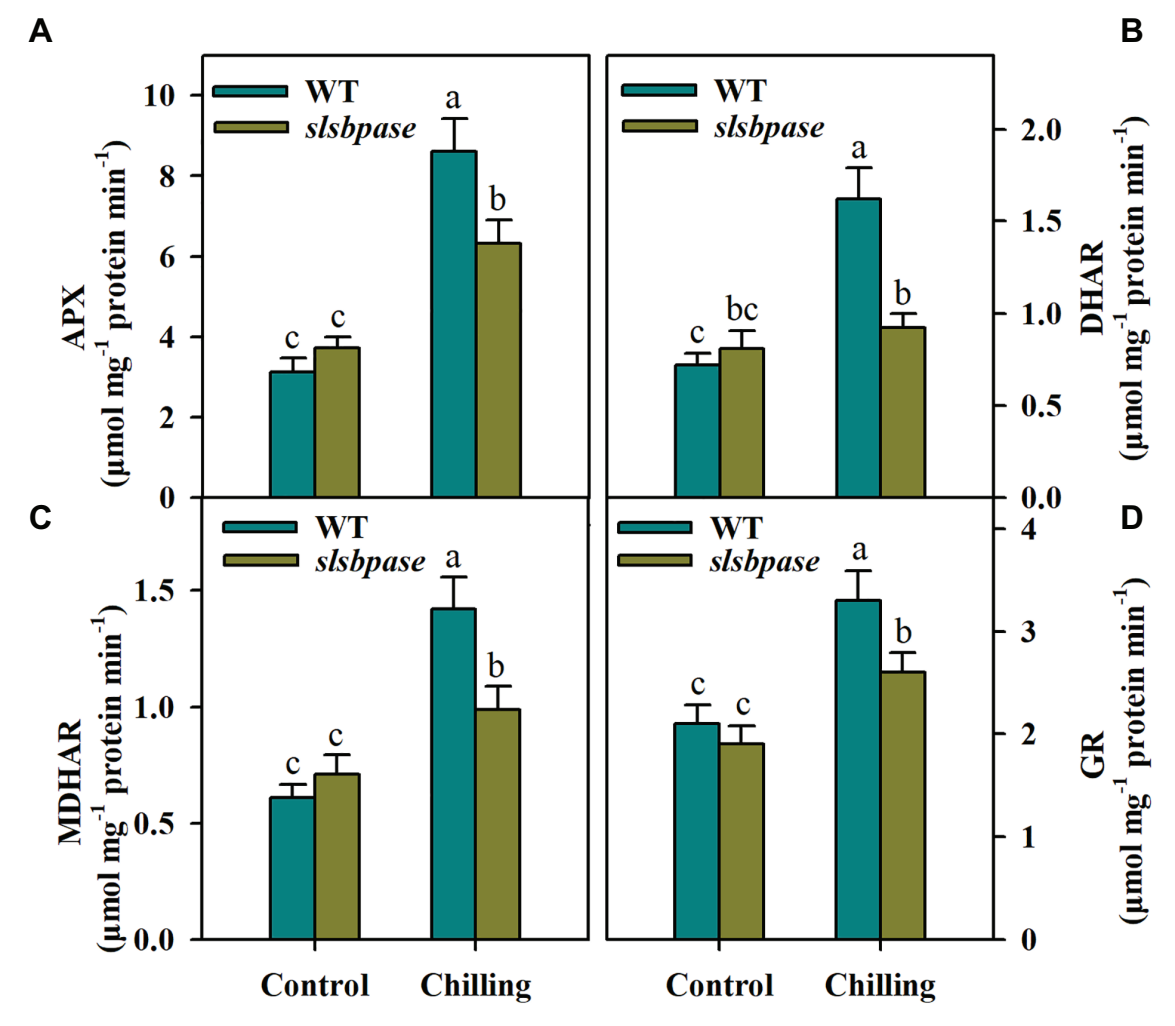
Activities of enzymes involved in the AsA-GSH pathway as affected by *SlSBPASE* mutation in chilling-stressed tomato plants. **(A)** Ascorbate peroxidase (APX); **(B)** dehydroascorbate reductases (DHAR); **(C)** monodehydroascorbate reductase (MDHAR); and **(D)** glutathione reductases (GR). At the fourth-leaf stage, *slsbpase* mutants and wild-type plants were subjected to a 24 h chilling stress. Following that, leaves from different groups were sampled for the determination of enzyme activities. The results are mean values ± SDs from three independent experiments, and different letters on top of each column represent significantly different values at *p* < 0.05 among treatments.

The interconversions between reduced and oxidized AsA and GSH involve several enzymes, including APX, DHAR, MDHAR, and GR. These enzymes displayed similar changes in activities in response to chilling temperature. In contrast to tomato plants under control conditions, those subjected to chilling stress exhibited higher activities of AsA-GSH cycle enzymes. However, under chilling stress, the activities of the four enzymes were significantly reduced in *slsbpase* mutants in comparison of those measured in wild-type plants (Figures 5A–D). These results suggest that *SlSBPASE* mutation inhibits recycling of AsA and GSH in chilling-stressed tomato plants.

### Mutation in *SlSBPASE* Suppresses Activities of Glutathione Biosynthetic Enzymes and Reduces Transcript Abundance of Their Coding Genes

**Figure 6:**
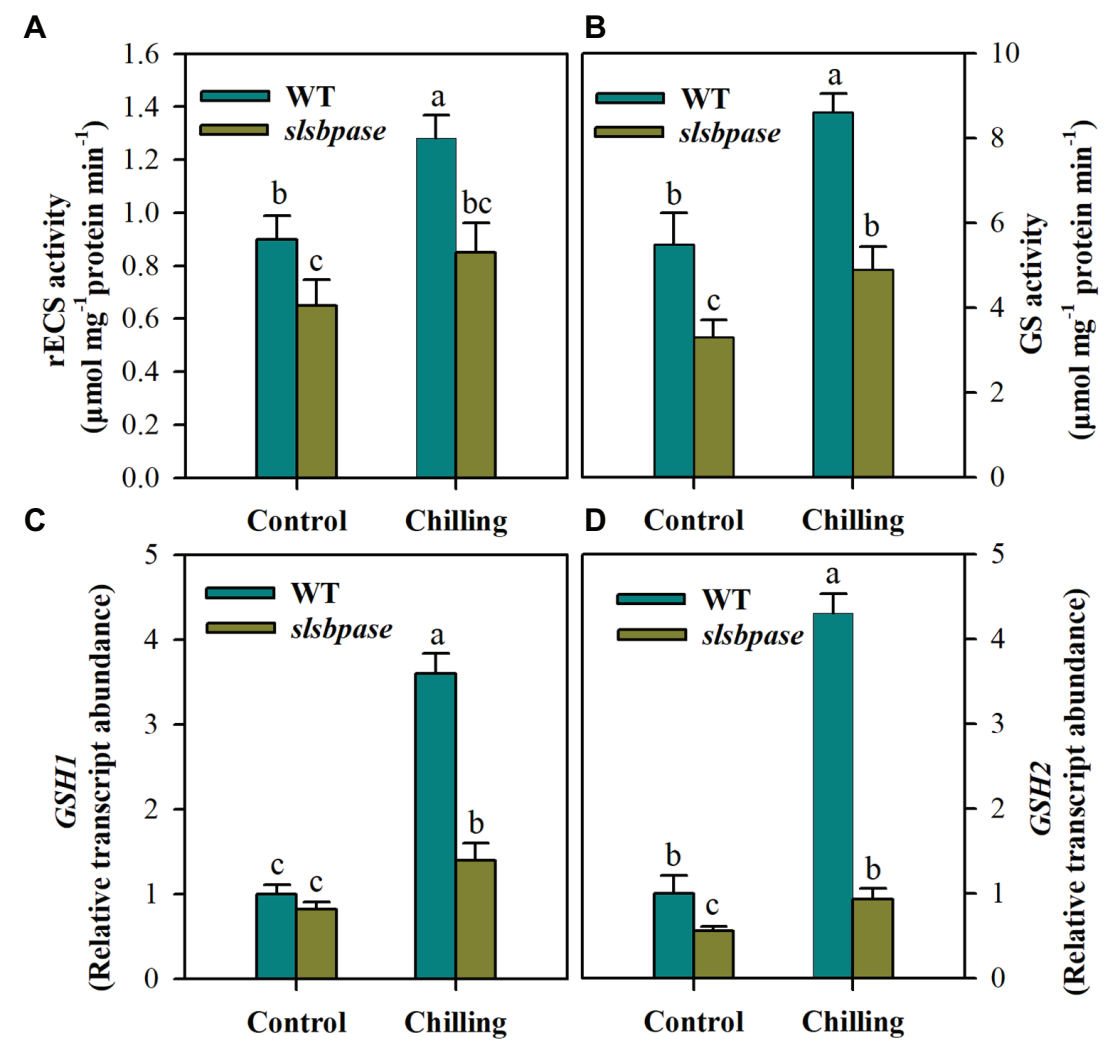
Activities of gamma-glutamylcysteine synthetase (γ-ECS; **A**) and glutathione synthetase (GS; **B**) in the pathway of GSH biosynthesis and relative transcript abundance of *GSH1* **(C)** and *GSH2* **(D)**. At the fourth-leaf stage, *slsbpase* mutants and wild-type plants were subjected to a 24 h chilling stress. Following that, leaves from different groups were sampled for the determination of enzyme activities and transcript abundance. The expression level in wild-type leaves under control conditions was set to 1, and the relative expression level in the rest of samples was calculated accordingly. The results are mean values ± SDs from three independent experiments, and different letters on top of each column represent significantly different values at *p* < 0.05 among treatments.

Having found that total GSH was remarkably decreased in *slsbpase* mutant plants under chilling stress, we next examined the changes in activities of two enzymes, including γ-ECS and GS, involved in GSH synthesis. The activity of γ-ECS showed significant difference between wild-type plants and *slsbpase* mutant plants under the control condition, and the difference was even larger under the chilling condition, with the activity being substantially reduced in *slsbpase* plants compared with that in wild-type plants (Figure 6A). Similar results were also observed for GS activity in both genotypes of plants (Figure 6B). Further examination of transcript abundance of *GSH1* and *GSH2*, encoding γ-ECS and GS, respectively, showed that mutation in *SlSBPASE* decreased the expression of both genes under control and chilling conditions (Figures 6C,D). These results demonstrate that loss of SBPase in tomato plants negatively affects GSH biosynthesis.

### Exogenous Application of GSH Reduces Chilling Sensitivity of *slsbpase* Mutant Plants

**Figure 7:**
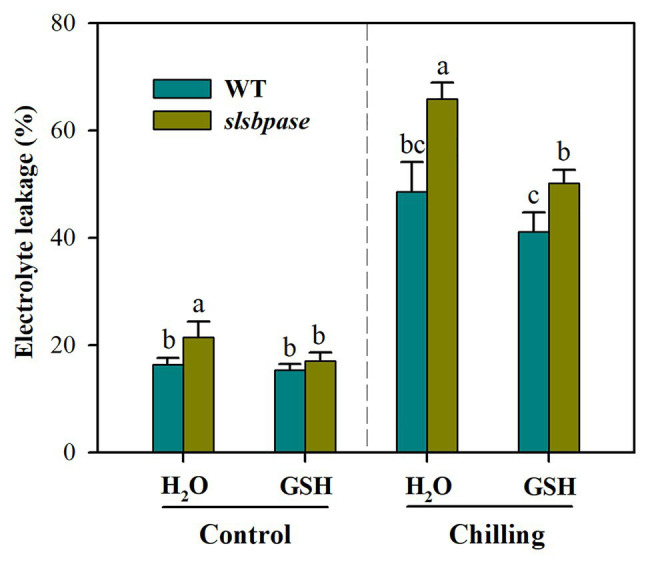
Effects of exogenous GSH on chilling damage as represented by relative electrolyte leakage. At the fourth-leaf stage, *slsbpase* mutants and wild-type plants were sprayed with or without GSH on 3 consecutive days and then subjected to a 24 h chilling stress. Following that, leaves from different groups were sampled for the determination of electrolyte leakage. The results are mean values ± SDs from three independent experiments, and different letters on top of each column represent significantly different values at *p* < 0.05 among treatments.

To further verify that decreased GSH contributes to increased chilling damage in *slsbpase* mutant plants, we performed a GSH feeding experiment. It was observed that exogenous application of GSH significantly reduced chilling damage of both wild-type and mutant plants as represented by decreased electrolyte leakage (Figure 7). Further analysis showed that without GSH application, electrolyte leakage was increased by 35.6% in *slsbpase* plants relative to that in wild-type plants under chilling stress; however, with GSH application, the increase was by 21.9%. These results indicate that exogenous GSH contributes more to the alleviation of chilling damage in *slsbpase* plants than in wild-type plants, and imply that decreased GSH as a result of *SlSBPASE* mutation may explain, in part, the increased chilling sensitivity of tomato plants.

## Discussion

SBPase functions in the essential photosynthetic carbon assimilation pathway, Calvin-Benson cycle, in C3 plants. SBPase catalyzes the production of sedoheptulose-7-phosphate, which is further utilized to regenerate RuBP, the CO_2_ acceptor required for the initiation of carbon fixation. Previous studies using transgenic lines of model and crop species have highlighted the importance of SBPase in photosynthetic carbon fixation and growth (Harrison et al., 2001; Lefebvre et al., 2005; Lawson et al., 2006; Rosenthal et al., 2011). Early work has shown that SBPase is also involved in the protection of plants against salt and high temperature stresses (Feng et al., 2007a,b). In our previous study, we found that overexpression of SBPase confers chilling tolerance in tomato plants (Ding et al., 2017c). However, how SBPase acts in the improvement of chilling tolerance remains largely unexplored. Previously, we have generated mutagenesis in the gene encoding SBPase using CRISPR/Cas9 technology in tomato plants and obtained *slsbpase* mutant plants (Ding et al., 2018a). In this study, comparative studies were made between *slsbpase* plants and wild-type plants under chilling conditions, which enable us to uncover the role of SBPase in chilling tolerance in tomato plants. We have concluded that SBPase is required for tomato plants to optimally respond to low temperature stress and the evidence includes (1) mutation in *SlSBPASE* increased ROS production and exacerbated lipid peroxidation in chilling-stressed tomato plants, (2) mutation in *SlSBPASE* negatively affected the AsA-GSH cycle under chilling stress, (3) mutation in *SlSBPASE* impairs GSH biosynthesis in chilling-stressed tomato plants, and (4) exogenous GSH partially recovers chilling resistance of *slsbpase* mutant plants.

Harsh environmental conditions, such as low temperature and drought, threaten metabolic processes and disrupt cellular homeostasis in plants. One of the unwanted but inevitable outcomes of environmental stress is the excessive accumulation of ROS, which causes oxidative damages in plants. In the present work, we observed that exposure to chilling substantially increased production of H_2_O_2_ and O_2_·^−^ in both mutant and wild-type tomato plants. However, the levels of ROS in *slsbpase* mutant plants were significantly higher than those in wild-type plants, suggesting that mutation in *SlSBPASE* increases ROS production. Photosynthesis takes place in chloroplast, which serves as a major site of ROS production (Allen and Ort, 2001). Under optimal growth conditions, a balance exists between the excitation of photosystems and the electron consumption; however, under stress conditions, energy consumption is markedly inhibited and surplus energy is passed on to molecular oxygen, giving rise to ROS. In this study, *slsbpase* mutant plants showed severe inhibition of carbon assimilation, which may decrease energy consumption and speed up ROS production. Thus, the difference in the capacity for carbon fixation between wild-type and *slsbpase* mutant plants may partly explain the discrepancy of ROS accumulation between these two groups of plants under chilling stress.

Under stress conditions, plants rely on a coordinated antioxidant defense network to remove excessive ROS. Antioxidant enzymes are important components of this network. In this study, we observed that chilling stress boosted activities of SOD, CAT, POD, and APX in both *slsbpase* mutants and wild-type plants. Surprisingly, no significant differences in SOD and CAT activities were observed between mutant and wild-type plants under chilling conditions. These results suggest that loss-of-function of SBPase does not exert influence on antioxidant machinery involving SOD and CAT and the observed increase in ROS accumulation in *slsbpase* mutant plants may result from disruption of another antioxidant machinery. In an attempt to uncover potential antioxidant system that may be impaired by mutation of SlSBPASE, we conducted a thorough analysis of the AsA-GSH cycle, which is a well-known antioxidant machinery in different organisms.

The AsA-GSH cycle plays a pivotal role in the removal of excessive ROS, protecting plants against oxidative stress triggered by adverse growth conditions (Hasanuzzaman et al., 2019). The cycle is comprised of AsA, GSH, and four enzymes, including APX, MDHAR, DHAR, and GR. Mutation in *SlSBPASE* significantly downsized the pools of total AsA and GSH, as well as their respective reduced forms in chilling-stressed tomato plants. Because AsA and GSH act as antioxidants and are of paramount importance in regulating redox balance in plants, maintaining their pools is crucial for the alleviation of oxidative stress. Hence, the observed decrease in AsA and GSH pools is in concert with the increased ROS accumulation in *slsbpase* mutant plants, suggesting the importance of SBPase in the control of ROS production through the AsA-GSH cycle.

However, mechanisms of SBPase influencing AsA and GSH pools remain unknown. In plants, AsA biosynthesis occurs mainly *via* Smirnoff-Wheeler pathway, which uses glucose derivative as the initial substrate (Wheeler et al., 1998). We observed that mutation in *SlSBPASE* resulted in a remarkable decrease in sucrose in tomato plants (Figure 1B), which may lead to the reduction of glucose in *slsbpase* mutant plants. It is thus speculated that the decreased sucrose due to SBPase knockout may contribute to the decreased AsA pool we observed in this study. The pathway of GSH biosynthesis in plants has been well established. GSH biosynthesis is a two-step ATP-dependent process, which involves two major enzymes. One enzyme is γ-ECS, catalyzing the formation of γ-glutamylcysteine from glutamate and cysteine and the other is GS, responsible for the addition of a glycine residue to γ-glutamylcysteine to produce GSH. We found that mutation in *SlSBPASE* led to the suppression of activities of γ-ECS and GS, as well as the transcription of their coding genes, *GSH1* and *GSH2*. As both γ-ECS and GS are key GSH biosynthetic enzymes, this result partly explains the decreased GSH pool in *slsbpase* mutant plants. We previously found that knockout of *SlSBPASE* dramatically alters nitrogen metabolism and reduces overall amino acids (Ding et al., 2018a). It is, therefore, reasoned that mutation in *SlSBPASE* may also negatively affect GSH biosynthesis by curtailing the levels of three essential amino acids for GSH.

Considering the importance of GSH in the alleviation of oxidative stress and the disrupted GSH biosynthesis in *slsbpase* mutant plants, we speculate that the increase in chilling sensitivity of tomato plants due to mutation of *SlSBPASE* may be partially ascribed to reduced GSH. In a GSH feeding experiment, we found that exogenous GSH significantly mitigated the chilling damage to tomato plants and the mitigation was more pronounced in *slsbpase* plants than that in wild-type plants, providing additional evidence that the reduced GSH level as a consequence of *SlSBPASE* mutation contributes to the increased chilling sensitivity in tomato plants.

The ratios of AsA/DHA and GSH/GSSG determine the redox potentials of AsA and GSH. Relative to DHA and GSSH, higher levels of AsA and GSH are important for ROS detoxification in plants under stress conditions. AsA and GSH are connected in the AsA-GSH pathway and are recycled *via* four enzymes, including APX, MDHAR, DHAR, and GR. Using reducing power from AsA, APX is capable of efficiently catalyzing H_2_O_2_ into water and monodehydroascorbate (MDHA), which is further converted to dehydroascorbate (DHA) by MDHAR. AsA is regenerated from its oxidized form DHA by DHAR and this reaction requires GSH as reducing agent (Soares et al., 2019). Another key enzyme in the AsA-GSH cycle is GR, which recycles GSSG back to GSH, allowing to maintain a high ratio of GSH/GSSG. As such, sustaining high activities of these enzymes accomplishes high ratios of AsA/DHA and GSH/GSSG. Our results showed that mutation in *SlSBPASE* significantly decreased activities of the four enzymes, which may ultimately lead to the decline in AsA and GSH as well as ratios of AsA/DHA and GSH/GSSG. These results partly explain the increased ROS accumulation in *slsbpase* mutant plants and substantiate the idea that SBPase favors removal of ROS *via* the AsA-GSH cycle.

**Figure 8:**
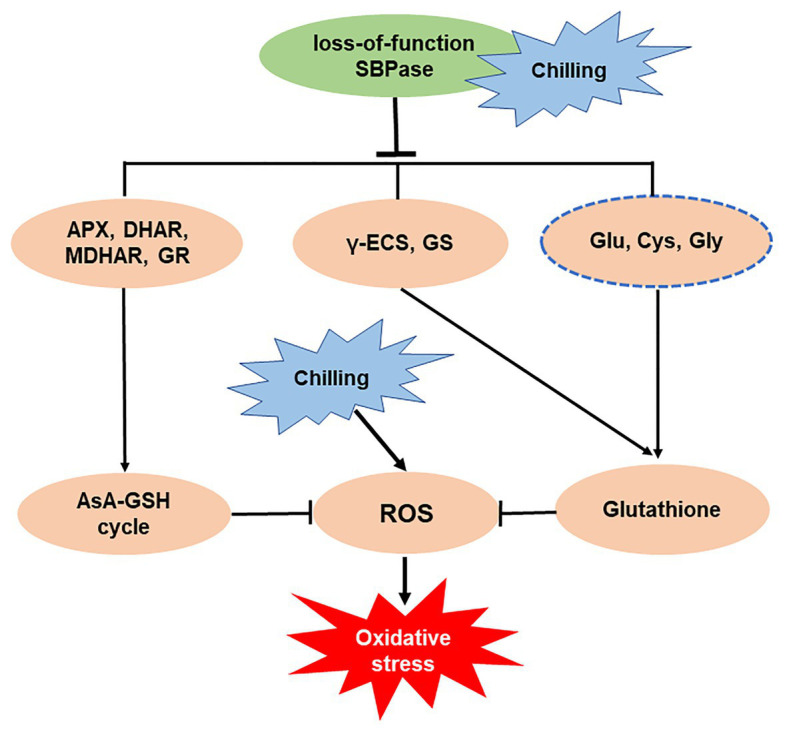
Simplified schematic representation of the importance of sedoheptulose-1,7-bisphosphatase (SBPase) in the mitigation of oxidative stress triggered by low temperature.

In summary, our study has demonstrated a central role of SBPase in the mitigation of oxidative stress triggered by low temperature in tomato plants. Mutation in *SlSBPASE* aggravates chilling-induced accumulation of ROS through impaired GSH biosynthesis and suppressed AsA-GSH recycling (Figure 8). Our previous study has revealed that overexpression of *SlSBPASE* enhances chilling-induced oxidative stress in tomato plants, however, the impact of *SlSBPASE* overexpression on GSH biosynthesis has not been investigated. Thus, future studies should explore the relationship between overexpressing *SlSBPASE* and GSH biosynthesis under cold stress. The results may help increase our understanding of the mode of action of *SlSBPASE* in the tolerance to cold-induced oxidative stress in plants.

## Data Availability Statement

The original contributions presented in the study are included in the article/supplementary material and further inquiries can be directed to the corresponding authors.

## Author Contributions

SZ and FD designed the study. MW and FD performed the experiments and analyzed the data. MW, SZ, and FD discussed the results. MW drafted the manuscript and FD and SZ revised it. All authors contributed to the article and approved the submitted version.

### Conflict of Interest

The authors declare that the research was conducted in the absence of any commercial or financial relationships that could be construed as a potential conflict of interest.

## References

[ref1] Allen D. J. Ort D. R. (2001). Impacts of chilling temperatures on photosynthesis in warm-climate plants. Trends Plant Sci. 6, 36–42. 10.1016/S1360-1385(00)01808-2, PMID: 11164376

[ref2] Apel K. Hirt H. (2004). Reactive oxygen species: metabolism, oxidative stress, and signal transduction. Annu. Rev. Plant Biol. 55, 373–399. 10.1146/annurev.arplant.55.031903.141701, PMID: 15377225

[ref3] Asada K. (2006). Production and scavenging of reactive oxygen species in chloroplasts and their functions. Plant Physiol. 141, 391–396. 10.1104/pp.106.082040, PMID: 16760493 PMC1475469

[ref5] Beauchamp C. Fridovich I. (1971). Superoxide dismutase: improved assays and an assay applicable to acrylamide gels. Anal. Biochem. 44, 276–287. 10.1016/0003-2697(71)90370-8, PMID: 4943714

[ref6] Cakmak I. Marschner H. (1992). Magnesium deficiency and high light intensity enhance activities of superoxide dismutase, ascorbate peroxidase, and glutathione reductase in bean leaves. Plant Physiol. 98, 1222–1227. 10.1104/pp.98.4.1222, PMID: 16668779 PMC1080336

[ref7] Cobbett C. S. May M. J. Howden R. Rolls B. (1998). The glutathione-deficient, cadmium-sensitive mutant, cad2-1, of *Arabidopsis thaliana* is deficient in γ-glutamylcysteine synthetase. Plant J. 16, 73–78. 10.1046/j.1365-313X.1998.00262.x, PMID: 9807829

[ref8] Ding F. Hu Q. Wang M. Zhang S. (2018a). Knockout of *SlSBPASE* suppresses carbon assimilation and alters nitrogen metabolism in tomato plants. Int. J. Mol. Sci. 19:4046. 10.3390/ijms19124046, PMID: 30558146 PMC6320769

[ref9] Ding F. Liu B. Zhang S. (2017a). Exogenous melatonin ameliorates cold-induced damage in tomato plants. Sci. Hortic. 219, 264–271. 10.1016/j.scienta.2017.03.029

[ref10] Ding F. Wang M. Liu B. Zhang S. (2017b). Exogenous melatonin mitigates photoinhibition by accelerating non-photochemical quenching in tomato seedlings exposed to moderate light during chilling. Front. Plant Sci. 8:244. 10.3389/fpls.2017.00244, PMID: 28265283 PMC5316535

[ref11] Ding F. Wang M. Zhang S. (2017c). Overexpression of a Calvin cycle enzyme SBPase improves tolerance to chilling-induced oxidative stress in tomato plants. Sci. Hortic. 214, 27–33. 10.1016/j.scienta.2016.11.010

[ref12] Ding F. Wang M. Zhang S. (2018b). Sedoheptulose-1,7-bisphosphatase is involved in methyl jasmonate‐ and dark-induced leaf senescence in tomato plants. Int. J. Mol. Sci. 19:3673. 10.3390/ijms19113673, PMID: 30463360 PMC6275076

[ref13] Ding F. Wang M. Zhang S. Ai X. (2016). Changes in SBPase activity influence photosynthetic capacity, growth, and tolerance to chilling stress in transgenic tomato plants. Sci. Rep. 6:32741. 10.1038/srep32741, PMID: 27586456 PMC5009361

[ref14] Driever S. M. Simkin A. J. Alotaibi S. Fisk S. J. Madgwick P. J. Sparks C. A.. (2017). Increased SBPase activity improves photosynthesis and grain yield in wheat grown in greenhouse conditions. Philos. Trans. R. Soc. B Biol. Sci. 372:20160384. 10.1098/rstb.2016.0384, PMID: 28808101 PMC5566882

[ref15] Feng L. Han Y. Liu G. An B. Yang J. Yang G.. (2007a). Overexpression of sedoheptulose-1, 7-bisphosphatase enhances photosynthesis and growth under salt stress in transgenic rice plants. Funct. Plant Biol. 34, 822–834. 10.1071/FP07074, PMID: 32689410

[ref16] Feng L. Wang K. Li Y. Tan Y. Kong J. Li H.. (2007b). Overexpression of SBPase enhances photosynthesis against high temperature stress in transgenic rice plants. Plant Cell Rep. 26, 1635–1646. 10.1007/s00299-006-0299-y, PMID: 17458549

[ref17] Foyer C. H. Halliwell B. (1976). The presence of glutathione and glutathione reductase in chloroplasts: a proposed role in ascorbic acid metabolism. Planta 133, 21–25. 10.1007/BF00386001, PMID: 24425174

[ref18] Foyer C. H. Shigeoka S. (2011). Understanding oxidative stress and antioxidant functions to enhance photosynthesis. Plant Physiol. 155, 93–100. 10.1104/pp.110.166181, PMID: 21045124 PMC3075779

[ref19] Gill S. S. Tuteja N. (2010). Reactive oxygen species and antioxidant machinery in abiotic stress tolerance in crop plants. Plant Physiol. Biochem. 48, 909–930. 10.1016/j.plaphy.2010.08.016, PMID: 20870416

[ref20] Griffith O. W. (1980). Determination of glutathione and glutathione disulfide using glutathione reductase and 2-vinylpyridine. Anal. Biochem. 106, 207–212. 10.1016/0003-2697(80)90139-6, PMID: 7416462

[ref21] Harrison E. P. Olcer H. Lloyd J. C. Long S. P. Raines C. A. (2001). Small decreases in SBPase cause a linear decline in the apparent RuBP regeneration rate, but do not affect Rubisco carboxylation capacity. J. Exp. Bot. 52, 1779–1784. 10.1093/jexbot/52.362.1779, PMID: 11520866

[ref22] Hasanuzzaman M. Borhannuddin Bhuyan M. H. M. Anee T. I. Parvin K. Nahar K. Al Mahmud J.. (2019). Regulation of ascorbate-glutathione pathway in mitigating oxidative damage in plants under abiotic stress. Antioxidants 8:384. 10.3390/antiox8090384, PMID: 31505852 PMC6770940

[ref23] Hussain H. A. Hussain S. Khaliq A. Ashraf U. Anjum S. A. Men S.. (2018). Chilling and drought stresses in crop plants: implications, cross talk, and potential management opportunities. Front. Plant Sci. 9:393. 10.3389/fpls.2018.00393, PMID: 29692787 PMC5902779

[ref24] Hutchison R. S. Groom Q. Ort D. R. (2000). Differential effects of chilling-induced photooxidation on the redox regulation of photosynthetic enzymes. Biochemistry 39, 6679–6688. 10.1021/bi0001978, PMID: 10828986

[ref25] Jabs T. Dietrich R. A. Dang J. L. (1996). Initiation of runaway cell death in an *Arabidopsis* mutant by extracellular superoxide. Science 273, 1853–1856. 10.1126/science.273.5283.1853, PMID: 8791589

[ref26] Lawson T. Bryant B. Lefebvre S. Lloyd J. C. Raines C. A. (2006). Decreased SBPase activity alters growth and development in transgenic tobacco plants. Plant Cell Environ. 29, 48–58. 10.1111/j.1365-3040.2005.01399.x, PMID: 17086752

[ref27] Lefebvre S. Lawson T. Zakhleniuk O. V. Lloyd J. C. Raines C. A. (2005). Increased sedoheptulose-1,7-bisphosphatase activity in transgenic tobacco plants stimulates photosynthesis and growth from an early stage in development. Plant Physiol. 138, 451–460. 10.1104/pp.104.055046, PMID: 15863701 PMC1104198

[ref28] Mittler R. (2002). Oxidative stress, antioxidants and stress tolerance. Trends Plant Sci. 7, 405–410. 10.1016/S1360-1385(02)02312-9, PMID: 12234732

[ref29] Nakano Y. Asada K. (1981). Hydrogenperoxide is scavenged by ascorbate-specific peroxidase in spinach chloroplasts. Plant Cell Physiol. 22, 867–880. 10.1093/oxfordjournals.pcp.a076232

[ref30] Nickel K. S. Cunningham B. A. (1969). Improved peroxidase assay method using leuco 2,3',6-trichloroindophenol and application to comparative measurements of peroxidatic catalysis. Anal. Biochem. 27, 292–299. 10.1016/0003-2697(69)90035-9, PMID: 5767184

[ref31] Olçer H. Lloyd J. C. Raines C. A. (2001). Photosynthetic capacity is differentially affected by reductions in sedoheptulose-1,7-bisphosphatase activity during leaf development in transgenic tobacco plants. Plant Physiol. 125, 982–989. 10.1104/pp.125.2.982, PMID: 11161054 PMC64898

[ref33] Patterson B. D. MacRae E. A. Ferguson I. B. (1984). Estimation of hydrogen peroxide in plant extracts using titanium(IV). Anal. Biochem. 139, 487–492. 10.1016/0003-2697(84)90039-3, PMID: 6476384

[ref34] Raines C. A. Harrison E. P. Lloyd J. C. (2000). Investigating the role of the thiol-regulated enzyme sedoheptulose-1, 7-bisphosphatase in the control of photosynthesis. Physiol. Plant. 110, 303–308. 10.1111/j.1399-3054.2000.1100303.x

[ref35] Rosenthal D. M. Locke A. M. Khozaei M. Raines C. A. Long S. P. Ort D. R. (2011). Over-expressing the C3 photosynthesis cycle enzyme sedoheptulose-1-7 bisphosphatase improves photosynthetic carbon gain and yield under fully open air CO2 fumigation (FACE). BMC Plant Biol. 11:123. 10.1186/1471-2229-11-123, PMID: 21884586 PMC3185276

[ref36] Rüegsegger A. Brunold C. (1992). Effect of cadmium on γ-glutamylcysteine synthesis in maize seedlings. Plant Physiol. 99, 428–433. 10.1104/pp.99.2.428, PMID: 16668902 PMC1080479

[ref37] Sarker U. Oba S. (2018). Catalase, superoxide dismutase and ascorbate-glutathione cycle enzymes confer drought tolerance of *Amaranthus tricolor*. Sci. Rep. 8:16496. 10.1038/s41598-018-34944-0, PMID: 30405159 PMC6220278

[ref38] Shi Q. Ding F. Wang X. Wei M. (2007). Exogenous nitric oxide protect cucumber roots against oxidative stress induced by salt stress. Plant Physiol. Biochem. 45, 542–550. 10.1016/j.plaphy.2007.05.005, PMID: 17606379

[ref39] Soares C. Carvalho M. E. A. Azevedo R. A. Fidalgo F. (2019). Plants facing oxidative challenges—a little help from the antioxidant networks. Environ. Exp. Bot. 161, 4–25. 10.1016/j.envexpbot.2018.12.009

[ref40] Stitt M. Lilley R. Gerhardt R. Heldt H. W. (1989). “Metabolite levels in specific cells and subcellular compartments of plant leaves” in Methods in enzymology. eds. Fleischer S. Fleischer B. (Amsterdam: Academic Press), 518–552.

[ref41] Szarka A. Tomasskovics B. Bánhegyi G. (2012). The ascorbate-glutathione-α-tocopherol triad in abiotic stress response. Int. J. Mol. Sci. 13, 4458–4483. 10.3390/ijms13044458, PMID: 22605990 PMC3344226

[ref42] Wang M. Zhang T. Ding F. (2019). Exogenous melatonin delays methyl jasmonate-triggered senescence in tomato leaves. Agronomy 9:795. 10.3390/agronomy9120795

[ref43] Wang M. Zhang S. Ding F. (2020). Melatonin mitigates chilling-induced oxidative stress and photosynthesis inhibition in tomato plants. Antioxidants 9:218. 10.3390/antiox9030218, PMID: 32155702 PMC7139585

[ref44] Wheeler G. L. Jones M. A. Smirnoff N. (1998). The biosynthetic pathway of vitamin C in higher plants. Nature 393, 365–369. 10.1038/30728, PMID: 9620799

[ref45] Yarmolinsky D. Brychkova G. Kurmanbayeva A. Bekturova A. Ventura Y. Khozin-Goldberg I.. (2014). Impairment in sulfite reductase leads to early leaf senescence in tomato plants. Plant Physiol. 165, 1505–1520. 10.1104/pp.114.241356, PMID: 24987017 PMC4119034

[ref46] Zushi K. Kajiwara S. Matsuzoe N. (2012). Chlorophyll a fluorescence OJIP transient as a tool to characterize and evaluate response to heat and chilling stress in tomato leaf and fruit. Sci. Hortic. 148, 39–46. 10.1016/j.scienta.2012.09.022

